# Evaluation of an online text simplification editor using manual and automated metrics for perceived and actual text difficulty

**DOI:** 10.1093/jamiaopen/ooac044

**Published:** 2022-05-30

**Authors:** Gondy Leroy, David Kauchak, Diane Haeger, Douglas Spegman

**Affiliations:** 1 Management Information Systems, Eller College of Management, University of Arizona, Tucson, Arizona, USA; 2 Computer Science, Pomona College, Claremont, California, USA; 3 El Rio Community Health Center, Tucson, Arizona, USA

**Keywords:** text simplification, text difficulty, health literacy, user study, metrics

## Abstract

**Objective:**

Simplifying healthcare text to improve understanding is difficult but critical to improve health literacy. Unfortunately, few tools exist that have been shown objectively to improve text and understanding. We developed an online editor that integrates simplification algorithms that suggest concrete simplifications, all of which have been shown individually to affect text difficulty.

**Materials and Methods:**

The editor was used by a health educator at a local community health center to simplify 4 texts. A controlled experiment was conducted with community center members to measure perceived and actual difficulty of the original and simplified texts. Perceived difficulty was measured using a Likert scale; actual difficulty with multiple-choice questions and with free recall of information evaluated by the educator and 2 sets of automated metrics.

**Results:**

The results show that perceived difficulty improved with simplification. Several multiple-choice questions, measuring actual difficulty, were answered more correctly with the simplified text. Free recall of information showed no improvement based on the educator evaluation but was better for simplified texts when measured with automated metrics. Two follow-up analyses showed that self-reported education level and the amount of English spoken at home positively correlated with question accuracy for original texts and the effect disappears with simplified text.

**Discussion:**

Simplifying text is difficult and the results are subtle. However, using a variety of different metrics helps quantify the effects of changes.

**Conclusion:**

Text simplification can be supported by algorithmic tools. Without requiring tool training or linguistic knowledge, our simplification editor helped simplify healthcare related texts.

## Introduction

Simplifying healthcare and medical text to improve understanding is difficult. However, the broad use of text as a medium for information dissemination makes it a critical problem to address improve health literacy in general and more specifically increase how people understand, remember, and act upon the information. In the previous decade, several national programs have emphasized this goal and its importance. For example, the Affordable Care Act^1^ emphasizes patient-centeredness, the National Action Plan to Improve Health Literacy^2^ specifies national goals, and the Plain Writing Act^3^ demands clarity in government communications.^4^ These guidelines are helpful, but still require good writing skills and domain knowledge and they do not provide concrete suggestions for a given text.

Text simplification is difficult. Just like writing in general, writing good, simple text requires several skills that are difficult to replace by algorithms. There are no readily available tools to translate or improve an existing text or to support writing a new text. Most projects focus on applying a simplistic metric, such as a readability formula, to assign a difficulty label to a text. These do not guide the writer in a substantive manner. The readability formulas^5^^,^^6^ at best serve as stand-ins for complexity^7^ but little evidence exists for their actual effectiveness. For example, the Flesch-Kincaid formula is commonly used but its outcomes can be inconsistent^8^: few studies show a relationship with understanding, sometimes a weak relationship with perceived difficulty is found,^9^ but no correlation with the Cloze measure^10^ (ie, a fill-in-the-blanks test originally created to compare difficulty levels of texts), and they are insensitive to text cohesion.^11^^,^^12^ In a study focused entirely on perceived difficulty, Zheng and Yu^13^ found that the readability formulas correlated with each other, but not with reader perception of difficulty for either Wikipedia articles or electronic health records (EHR) notes. In our own work, we found that the formulas correlate with perceived difficulty, ie, how a text looks to a reader, but not with actual difficulty, ie, the understanding of the content reflected in the ability to answer questions about it.^14^

Recently, caution is being advised in the use of the formulas, for example, by the Centers for Medicare and Medicaid Services.^15^ Even so, the formulas are still often used to evaluate content. For example, several recent studies apply the formulas to specific online texts about topics such as COVID-19 health,^16^ laser resurfacing therapy,^17^ Trigeminal Neuralgia,^18^ tinnitus,^19^ and others. Other studies use formulas to provide reviews of sets of online resources, for example, of 157 online resources^20^ or 80 patient education materials.^21^ The general consensus is that the existing information is written at too high a readability level compared to the advised level of 6th to 8th grade. Unfortunately, most of these studies only apply formulas without involving representative readers to measure difficulty and comprehension.

On the other end of the spectrum, driven by recent advances in generative neural networks, a range of general-purpose automated simplification methods have been proposed. Al-Thanyyan and Azmi^22^ provide an overview of automated lexical and syntactic simplification approaches. Most of these algorithms train on aligned corpora of difficult and easy sentences (eg, English Wikipedia and Simple English Wikipedia^23^). Few systems have been evaluated on health-related or medical text since there is a lack of aligned medical text.^24^ Fully automated systems are usually evaluated using a combination of human and automated metrics that utilize gold standard text with known simplifications.^25^ The human metrics all measure different dimensions of perceived difficulty, ie, perceived simplicity, perceived grammaticality, and perceived information retention. Automated metrics compare the system output with the gold standard and attempt to measure overlap based on smaller text units, for example, the overlap of n-grams. The most common metrics are BLEU (ie, Bilingual Evaluation Understudy) and SARI (ie, System output Against References and Input sentence)^26^ and are derived from machine translation evaluation metrics, but additional metrics have been proposed.^25^ Recent neural network models are good at producing fluent text that improves the perceived difficulty of the text, however, information loss and errors are common and they are still not near the levels of human simplification.^22^ In our own work,^27^ we focused on an autocomplete tool that suggests a single word at a time to complete a sentence. Our best combination of neural models used an entire sentence as context and was able to suggest the correct, simple word (among 5) for 73% of the examples on a selection of medical texts extracted from Wikipedia.

Very few projects have undertaken the development of writer support tools and their evaluation with representative readers and writers. In our own work, we have applied large-scale studies, using both corpus statistics and machine learning, to discover individual features in text that indicate potential for directing simplification. These individual features are then evaluated with representative readers. Besides our own work, we found one example project that involved measuring comprehension by readers. He et al^28^ compared 3 simplifications of text with the original text: manual simplification, semi-automated lexical/syntactic simplification, and manually created visualization. Only the manual expert simplification as well as the manually created visualization yielded improvement in multiple-choice question answering.

For this work, we developed an online text editor for use by information providers that combines different types of simplification algorithms. Our guiding principle has been to include algorithms that have shown, using objective evaluations, to positively affect comprehension and retention of information or to be associated with known simpler text. Supplementary Appendix A lists our algorithms and associated studies in comparison to existing advice. We provide here an evaluation of our text editor using 4 texts that were rewritten by a health educator using the editor. They were read by members of a local community health center. Supplementary Appendix B contains the study materials: the original and simplified texts as well as the evaluation questions. In addition to questions for perceived and actual difficulty, we also gather free recall of information and use a human expert and an automated metric for evaluating this recall (ROUGE, a metric commonly used for evaluating summarization^29^).

## OBJECTIVE

Our objective is to assist text simplification with an experimentally validated, easy-to-use, online text editor. Our editor integrates a variety of text features (that have been shown to individually affect perceived difficulty [how difficult readers view the text] and actual difficulty [how the difficulty of the text affects understanding and comprehension]). The features are detected automatically in the text by underlying algorithms that highlight relevant sections of the text and provide suggestions for simplification. While the suggestions are automated, the editing process relies on a human-in-the loop to avoid the introduction of errors or omissions.

For this study, our goal was to evaluate our text editor with all of the features combined into a single tool. We conducted a study that focuses on the effects on readers. We measured perceived and actual difficulty of original and simplified texts with both manual and automated measures as part of a randomized user study. A representative health educator simplified the texts, and the study participants were recruited at a local community health center.

## MATERIALS AND METHODS

### Text simplification editor

**Figure 1.**
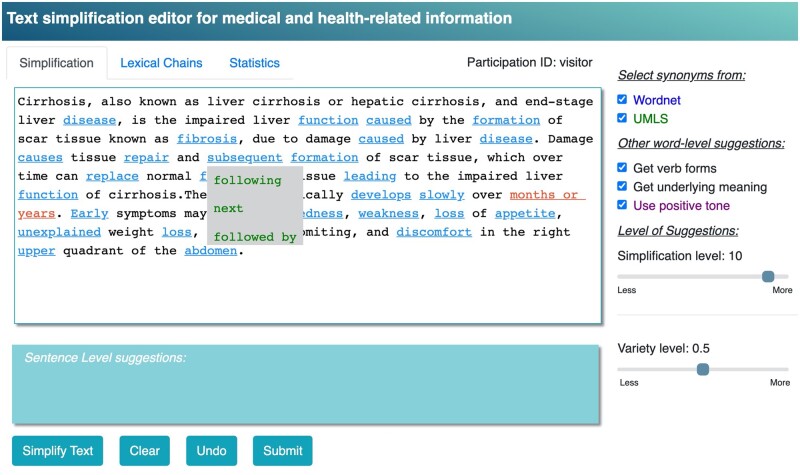
Screenshot of the editor for word level suggestions (by clicking “Simplify Text”). “Subsequent” has been clicked and potential substitutions are shown as a dropdown menu.

Figure 1 shows a screenshot of the editor being used on an excerpt from the cirrhosis Wikipedia page. The screen is divided into different sections along with 3 different tabs at the top. The main part of the tool is a text editor box, found under the “Simplification” tab. The user enters (eg, copy and pastes) the text into this text box and then clicks the “Simplify Text” button. The figure shows the results after the button has been clicked and the algorithmic feedback is shown.

Blue underlined words show words/phrases that have been identified as difficult and where the tool has simpler suggestions. These words/phrases can be clicked on to show the candidate suggestions as a dropdown menu. For example, there are 3 suggestions available for “subsequent”. These suggestions can be clicked on and the original word/phrase will be replaced in the text. The editor box is also a generic text editor and the text can be modified to fit appropriately, for example, if the user selected “followed by” as a replacement for “subsequent”, the writer might also choose to delete the preceeding “and”.

**Figure 2.**
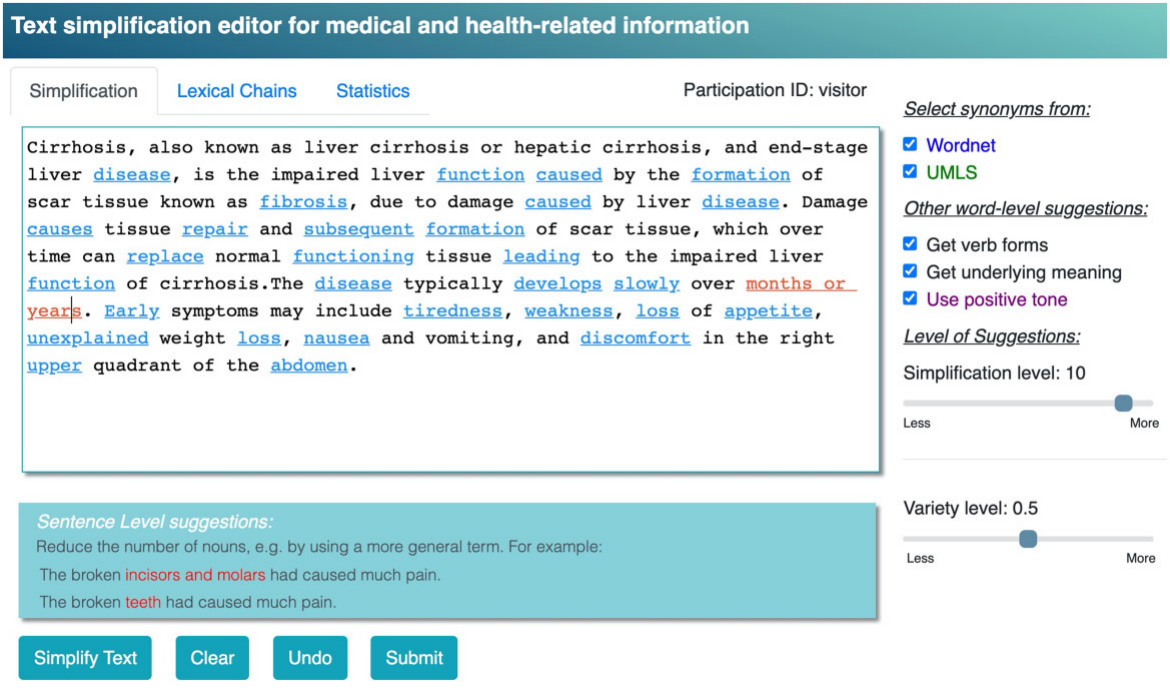
Screenshot of the editor for a sentence-level suggestion (“months or years”). The guidance with a new example, taken from a general corpus, is shown at the bottom in the colored box.

Sentence level feedback is show as underlined red sections. When one of these sections is clicked on, specific guidance on how to simplify the sentence structure is shown, including a concrete example. For example, Figure 2 shows the editor after “months or years” is clicked on and the guidance suggests to rephrase this structure (in particular, the portion of the sentence highlighted) along with an example. The examples provide additional guidance about how the rule can be used, but they are not adjusted per text and are not topic-specific. Existing corpora with multiple simplification levels are not yet available to generate topic-specific examples. Future editor versions will select examples that are matched to the text topic.

On the right side of the screen are various settings that can be adjusted. In the upper right are all of the word/phrase level resources that the editor uses. By default all are used, but the user may deselect any if they find that they are not helpful for their particular task and they will not be shown as suggestions. Additionally, there are 2 sliders that adjust thresholds. The “simplification level” enables adjustement of the number of words identified as difficult: decreasing it will show suggestions for fewer words. The “variety level” enables adjustment of the number of alternatives shown in the dropdown menu: decreasing it will show fewer suggestions with the selection. We tested and use our own word-embedding-based context filterer^30^ to select relevant suggestions.

**Figure 3.**
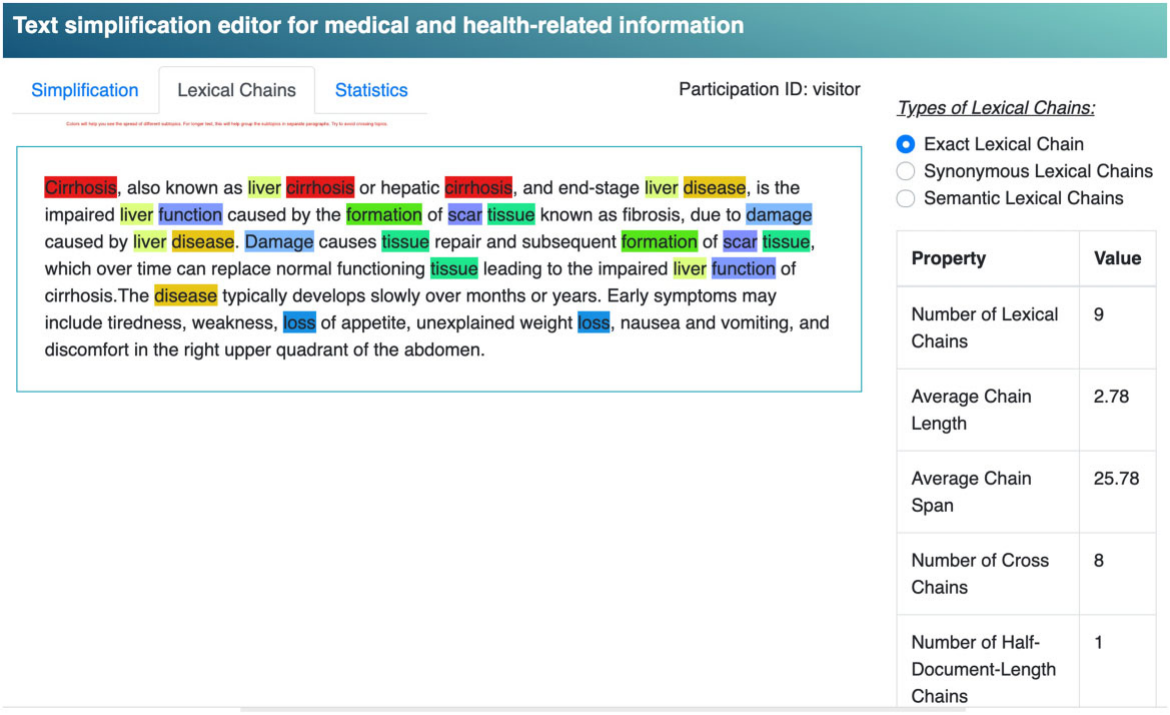
The lexical chains tab of the editor with the same text used in the previous 2 figures.

There are 2 additional tabs that can provide further useful information. Figure 3 shows the “Lexical Chains” tab. Different colors highlight different chains which correspond to themes of related words that occur throughout the text. The related statistics are shown on the right. These chains can help the user understand where clusters of information overlap and may need to be separated. Several lexical chain characteristics have been shown to relate to text difficulty, for example, there are more crossing chains in difficult text.^31^ The editor provides highlighting for 3 versions of lexical chains which recognize increasingly broader mapping of terms, ie, exact, synonymous, and semantic matching words.

The “Statistics” tab provides a comparison of various text statistic between the original input text and the final simplified text. These include the number of sentences, average sentence length, average word frequency (a measure of word difficulty), and counts for nouns and verbs.

Finally, on the server side, the tool records information to be used for future tool development, for example, the original and final simplified text, but also user activity, such as which words/phrases are clicked on and which candidate options are selected.

### Simplification process and texts

Four texts were simplified by a local health educator. Since we intend for the editor to be useful for any type of text, a variety of texts were included in our study: 2 long and 2 short texts on common or more exotic topics. Each text was originally available on Wikipedia and the topic and text segment were chosen so that common knowledge would be low. The 2 short texts covered pemphigus and polycythemia vera. The 2 longer texts covered asthma and liver cirrhosis. The educator was provided with a short verbal introduction of the functionality of our online editor. While they used her own judgment in simplifying the text, they were asked to use only the suggestions made by the editor and not to use outside sources, not to perform deletion of information, and not to include newly written summaries. No other directions were provided to ensure maximal external validity for the study. The questions asked about the content were not simplified. We attempted to use language that was not present in either the original or the simplified texts. Table 1 provides and overview of the original and simplified text characteristics. Supplementary Appendix B contains the texts and the questions posed to the participants.

**Table 1.** Text characteristics

	Long				Short			
	Asthma		Liver cirrhosis		Pemphigus		Polycythemia vera	
	Original	Simplified	Original	Simplified	Original	Simplified	Original	Simplified
Word count	623	626 (0.5)	481	517 (7.5)	198	177 (−10.6)	199	199 (0)
Sentences	31	34 (9.7)	25	26 (4.0)	15	14 (−6.7)	11	9 (−18.2)
Number of (exact) lexical chains	33	37 (12.1)	26	26 (0)	12	13 (8.3)	12	11 (−8.3)
Verb count	93	103 (10.8)	62	80 (29.0)	24	22 (−8.3)	22	25 (13.6)
Noun count	212	202 (−4.7)	180	184 (2.2)	67	64 (−4.5)	78	76 (−2.6)
Avg word frequency	280 834 273	327 044 769 (16.5)	313 157 350	347 837 287 (11.1)	325 116 425	393 023 079 (20.9)	276 426 270	286 907 512 (3.8)
Flesch-Kincaid	13.9	11.5 (−17.3)	14.5	12.7 (−12.4)	12.3	14.2 (15.4)	12.5	11.5 (−8.0)

### Study design

#### Participants

The study was conducted completely online in spring 2021 during the COVID-19 pandemic. Students of the “Weight Management for Optimal Health” class (approximately 90% female students) at the El Rio Community Health Center (Tucson, AZ) were invited to participate during their online class. Those who indicated interest to the instructor were sent a follow-up email with the study link. A few participants indicated interest by relatives and friends to participate in the study resulting in a snowball sample. These extra participants received their own email with their own study link. All participants were reimbursed for their time with $45 with an Amazon gift card or cash. The study was approved by the Institutional Review Board (IRB) of the University of Arizona.

#### Procedure

To ensure that the study could be completed in a timely manner and to avoid fatigue, we presented 4 texts to each participant: 2 in original and 2 in simplified format. To balance the order and the topics of texts, we created 6 combinations (combinations 1–6) each containing 4 texts (asthma, polycythemia vera, pemphigus, cirrhosis) in 1 of 2 versions (original, simplified) and randomized the order of the texts.

Participants who signed up for the study were assigned to 1 of the 6 combinations and provided with the related link. The study was conducted using REDCap surveys that contained a short introduction to the study, the 4 texts with questions, and a demographic survey.

#### Measures

We chose to include a variety of different measures to show a nuanced picture and to provide a background for comparison for future work. Perceived difficulty was measured with a 4-point Likert Scale (“After reading this text, I consider this information ….”) and 4 answer options “(Very difficult to understand,” “Difficult to understand,” “Easy to understand,” “Very easy to understand”), with the higher score (4) reflecting the easiest text.

Actual difficulty was measured with multiple-choice questions of which the choices were randomized per person. These questions focused on specific facts:

- Five true/false questions posed before and repeated after reading the text.
- Two multiple-choice questions with 4 choices presented while reading the text. They were easy to answer if the text was read completely and were included to encourage reading the entire text.
- Four multiple-choice questions with 4 choices presented after reading the text. They were more difficult to answer and required reading the text with attention.

We also used broader measures of actual difficulty based on free recall of information by the participants. We evaluate the information recalled by participants using manual and automated metrics:

- Double-blind evaluation by our health education expert who scored each answer for the number of correct facts, whether the main point was conveyed, correctness (scale: no errors (4), minor errors (3), major errors (2), mostly wrong (1), all wrong (0)), and completeness (scale: complete (4), most information (3), some items (2), minimal (1), nothing (0))
- Overall semantic match between the participants’ answers and the text was calculated using cosine similarity based on word embeddings. Word embeddings are semantic representations using vectors in a vector space. This allows calculation of similarity of words and texts without being restricted to exact matches or synonyms. We used Google’s Word2Vec pre- trained 300-dimension word embeddings.^32^ We calculate an overall similarity score, as well as the proportion of words in an answer that were similar or matching the original text.
- We also used automated metrics utilized to evaluate text summarization to capture the information recalled by the participants. We used ROUGE precision, recall, and F-Measure (ie, harmonic mean of the precision and recall). We calculated ROUGE scores for overlap of unigram, bigrams, and longest sequences.^29^

#### Data analysis

We performed one-way analysis of variance (ANOVA) using the text version with 2 conditions (original, simplified) as the independent variable. We report the main and standard deviation for each dependent variable together with the *F*-measure and *P*-value for the ANOVA. We performed these one-way ANOVAs for the perceived and actual difficulty measures.

## RESULTS

### Text characteristics

Table 1 shows an overview of the main characteristics of text before and after simplification. Overall, the changes made were subtle and difficult to quantify with simple metrics. For the longer texts, the word count slightly increased. The verb count also increased with slight decreases in noun count, which relates to some of our rules, for example, replacing nominalizations with verbs. The average word frequency also increased for the simplified texts, indicating use of more frequent (simpler) words.

### Participants

A total of 49 participants completed the study resulting in 196 texts being scored. To avoid analysis using data based on pure guessing or assumed knowledge without reading the text, we used the time spent to read the text as an indication of actual intent by participants. Data from submissions where <1 min was spent reading the text and the 2 accompanying multiple-choice questions was eliminated. One minute was determined as the cutoff because this was the fastest the authors could read the text and questions themselves. As a result, the scores for 41 texts were removed and 155 were retained. We believe this provides a balance that is not too lenient (ie, accept all data) or harsh (ie, remove all data if as few as 1 of 4 sections was finished too quickly).

The demographic information of the remaining 45 participants is shown in Table 2. The majority of the participants were female (87%). Participants could check multiple options for race and the majority identified as White (89%) with the next biggest group being American Indian or Alaska Native (11%). Almost half of the participants identified as Hispanic or Latino (42%). The education level was varied with the 2 largest groups having a bachelor’s degree as their highest degree (29%), followed by a high school diploma (24%) or master’s degree (24%). Only a few participants had no high school diploma (2%) or earned a doctorate (4%). All age levels were well represented with one small group of people 71 years or older (2%). All participants spoke at least some English at home, with most of them speaking only English (60%) or mostly English (31%).

**Table 2.** Participant demographic information

Variable	Choice	Count (%)
Sex		
	Female	39 (87)
	Male	6 (13)
Race (multiple options possible)		
	American Indian or Alaska Native	5 (11)
	Asian	1 (2)
	Black or African American	1 (2)
	Native Hawaiian or Other Pacific Islander	1 (2)
	White	40 (89)
Ethnicity		
	Hispanic or Latino	19 (42)
	Not Hispanic or Latino	26 (58)
Education level		
	Less than high school degree	1 (2)
	High school diploma	11 (24)
	Associate degree	7 (16)
	Bachelor’s degree	13 (29)
	Master’s degree	11 (24)
	Doctoral degree (PhD, MD, …)	2 (4)
Age		
	Younger than 30 years old	10 (22)
	31–40 years old	8(18)
	41–50 years old	9(20)
	51–60 years old	7 (16)
	61–70 years old	9 (20)
	71 years old or better	2 (2)
Language spoken at home		
	Never English	0 (0)
	Rarely English	1 (2)
	Half English	3 (7)
	Mostly English	14(31)
	Only English	27 (60)

### Perceived difficulty

We found a significant effect for perceived difficulty with simplified texts being seen as easier than the original text (see Table 3).

**Table 3.** ANOVA results for perceived difficulty (significant differences are in bold)

Metric	Condition		
	Original	Simplified	
	Mean (SD)	Mean (SD)	*F*-value/*P*-value
4-point Likert Scale	2.28 (.697)	2.55 (.681)	**6.130/.014**

### Actual difficulty

Table 4 shows the results for our measures focusing on specific content, overviews, and free recall measured with both manual and automated measures.

**Table 4.** ANOVA results for actual difficulty (significant differences are in bold and indicated by * for scores higher with simplified text and + for scores higher with original text)

Variable	Metric		Condition		
			Original	Simplified	
			Mean (SD)	Mean (SD)	*F*-value/*P*-value
Questions before reading	True/false (%)	TF1	67 (47)	63 (49)	0.261/.610
		TF2	71 (46)	71 (46)	0.001/.982
		TF3	51 (50)	53 (50)	0.061/.805
		TF4	76 (43)	72 (45)	0.257/.613
		TF5*	**33 (47)**	**59 (50)**	**11.450/.001**
Questions while reading	Multiple-choice (%)	Overview question	80 (79)	83 (76)	0.250/.618
		General question	71 (79)	70 (76)	0.024/.877
Questions after reading	True/false (%)	TF1	66 (48)	62 (49)	0.263/609
		TF2	93 (27)	88 (32)	0.793/.375
		TF3	66 (48)	72 (45)	0.770/.382
		TF4	93 (27)	92 (27)	0.005/.945
		TF5*	**52 (50)**	**78 (42)**	**11.921/.001**
	Multiple-choice (%)	MC1*	**37 (48)**	**57 (50)**	**6.320/.013**
		MC2	68 (47)	75 (44)	0.835/.362
		MC3*	**33 (47)**	**59 (50)**	**11.450/.001**
		MC4	33 (47)	29 (46)	0.282/.596
Automated/semantic evaluation	Unique word count (*N*)		25 (14)	23 (12)	0.891.347
	Proportion of words similar to text (%)*		**76 (16)**	**82 (11)**	**6.729/.010**
	Proportion of word matching to text (%)*		**53 (18)**	**59 (14)**	**5.248/.023**
	Overall cosine similarity*		**0.0.111009951634249 (.013)**	**0.116799197273372 (.009)**	**9.207/.003**
Manual/expert evaluation	Correct facts count (*N*)		7.5 (4.4)	8.1 (6.6)	0.357/.551
	Main point made (%)		67 (69)	67 (47)	0.016/.899
	Completeness (score)		2.10 (.89)	2.17 (.84)	0.145/.704
	Correctness (score)		3.18 (.86)	3.21 (.71)	0.058/.810
Automated/machine learning metric	Rouge recall—longest phrase		0.0930766 (0.06349999)	0.1078276 (0.06988539)	1.894/.171
	Rouge precision—longest phrase		0.4873848 (0.21197400)	0.4357437 (0.12496557)	3.380/.068
	Rouge F-measure—longest phrase		0.1514124 (0.09538131)	0.1637058 (0.09026941)	0.678.412
	Rouge recall—unigram*		**0.0850563 (0.05060304)**	**0.1186970 (0.08024499)**	**9.825/.002**
	Rouge precision—unigram		0.6893254 (0.15174880)	0.6535888 (0.13297372)	2.424/.122
	Rouge F-measure—unigram*		**0.1465184 (0.07850236)**	**0.1907154 (0.11077505)**	**8.263/.005**
	Rouge recall—bigram		0.0368748 (0.03753065)	0.0437070 (0.03943795)	1.221/.271
	Rouge precision—bigram+		**0.3156005 (0.26328089)**	**0.2401288 (0.14450423)**	**4.841/.029**
	Rouge F-measure—bigram		0.0639861 (0.06378179)	0.0699317 (0.05622001)	0.378/.540

The true/false question were provided before and after reading the text. There was one significant difference for question 5 before reading the text, with higher scores for the simplified text version (59%) versus the original version (33%).

The 2 multiple-choice questions presented with the text were answered with 80% and 83% accuracy for the overview question and 71% and 70 for the general questions. There was no significant difference between the 2 text versions.

There are more differences in the 4 multiple-choice questions presented after reading the text. For 2 questions accuracy was significantly higher with simplified text. For the first multiple-choice question, the simplified version leads to 20% higher accuracy (37% versus 57% for original and simplified texts) and for the third multiple-choice questions the difference is even larger with 26% difference in accuracy (33% versus 59% for original and simplified text). The second multiple-choice question shows a smaller, but not significant, increase.

The next set of metrics take a more global approach to evaluating the answers. They are based on the free recall of information. In general, there is no difference in answer length with 25 and 23 words written on average for recall of the original and simplified text.

The automated measures based on word embeddings show that overall, the recall of information is more similar to the information provided with the simplified text. There is a significant difference in the average cosine similarity with simplified text leading to a higher average cosine similarity (0.111 versus 0.117 cosine similarity for original and simplified text), and also a higher proportion of words in the answer that are semantically similar (76% versus 82% for original and simplified text) or that are an exact match (53% versus 59% for original and simplified text).

No significant differences were found based on the expert evaluation. The scores are slightly higher with simplified text, but not significant. Overall, 7.5 versus 8.1 facts were remembered for original and simplified text. The main point was made by about two-thirds of people (67% with both texts). The overall scores for correctness and completeness were almost the same with slightly lower scores for correctness than completeness.

To complete our evaluation of the information recalled, we calculated ROUGE scores. For unigrams, recall was significantly higher with simplified text. For bigrams, precision was significantly lower with simplified text.

### Secondary analysis

We conducted 2 exploratory follow-up analyses using the mean score of all multiple-choice questions with and after reading the text, ie, excluding questions before reading the text.

**Figure 4.**
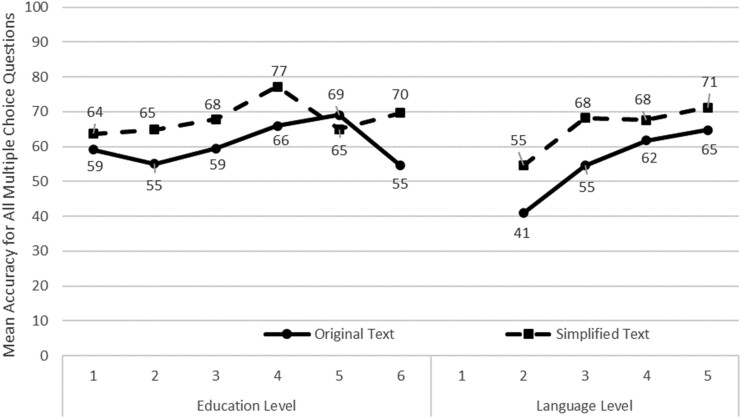
Mean accuracy answering questions by education and language level.

For the first analysis, we evaluated 2 types of self-reported demographic information: the education level and the language level. We calculated one-tailed Pearson Correlation coefficients. Overall, both the education level (*r* = .179, *P* = .013) and language level (*r* = .207, *P* = .005) are significantly correlated with the mean accuracy. Figure 4 shows an overview. This effect is present with the original text but disappears when the text is simplified. There is no significant correlation for education or language level and answering questions for the simplified texts. For the original text, the education level (*r* = .266, *P* = .009) and the language level (*r* = .563, *P* = .020) correlate with the mean accuracy. Given that these analyses are *posthoc*, a Bonferroni correction would require a significance level of 0.0125, making some correlations not statistically significant.

**Figure 5.**
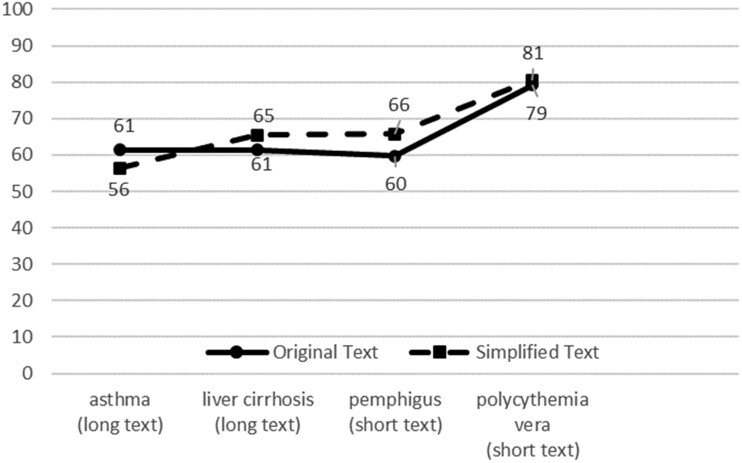
Mean accuracy answering questions per text.

Our second analysis focuses on the texts. Our goal is to create a simplification editor that is useful for a wide variety of texts. We therefore chose 4 texts with different characteristics and topics. Figure 5 shows the accuracy average over all questions averaged for the different texts. The topics being discussed in the text may not affect the editor’s effectiveness. While asthma may be considered the best-known topic, this is also where answers are lowest and do not improve with simplification. The low performance was due low scores on 2 of the 4 multiple-choice questions. However, simplified text led to overall improvements in 3 of the 4 texts with very different underlying characteristics.

## DISCUSSION

The design of the study focused on optimizing external validity with regard to readers: we included multiple readers and text that was simplified by a single writer. This approach allowed us to evaluate the effects of simplifying text and represents the situation where texts are optimized and then distributed to multiple patients or health information consumers. Our results show that even with limited training for the writer, the resulting simplified texts were generally better understood by readers. This was shown by 3 different measures: perceived difficulty as measured by the perception of the readers, actual difficulty as measured by answers to multiple-choice questions, and actual difficulty as measured by free recall of information. For perceived difficulty, simplified texts were perceived as significantly simpler as measured with a Likert-scale. For actual difficulty, all measures paint the same overall picture. Of the 4 multiple-choice questions, 3 showed higher answer accuracy with the simplified version and for 2 of them this difference was statistically significant. For true/false, 4 questions showed no significant difference, but for the fifth question (TF5) accuracy went from 52% for the original text (close to random) to 78% accuracy for the simplified text. The free recall of information by readers showed that their answers were more extensive and more related to the content with simplified text.

An unexpected finding was the significant difference for the fifth true/false question (TF5) before reading the text. We believe this may be due to the question being difficult and most people not answering it correctly before reading the text. When these questions were repeated after reading the text, scores went up. However, after reading the original text, the scores are still at approximately guessing level (52%) while after reading the simplified text, they are much higher (78%). It is note-worthy that creating objectives questions is difficult. When questions are easy, no differences in accuracy can be measured (ceiling effect). This may be reflected in the scores for questions posed together with the text that results in high accuracy (above 80%). When questions are difficult, a similar problem may exist (floor effect) and may explain the accuracy scores for the last question after reading the text which was close to guessing.

Our evaluation using free recall of information provides a more global picture than the multiple-choice questions. However, the answers were short, and this may have affected our automated measures. With short texts, there are more single words than multi-word phrases to base calculations on. This may explain why significant differences were only found using unigrams. Even so, the ROUGE scores indicate that recall is higher with simplified text and precision was lower. This is a trade-off commonly found with precision and recall measures.

We are considering several future, follow-up studies. First, we will focus on working with multiple writers and evaluating how they differ in their use of the tool. This approach will allow us to find gaps and opportunities for improvements based on different goals of the writers as well as different levels of expertise and medical knowledge of the writers. In addition, we may target different health conditions or treatments with different levels of difficulty required to provide an explanation. Finally, while we show in this study that our tool enabled lower educated individuals to answer questions equally well as those with higher education after simplification, we intend to evaluate more extensively the outcomes from using our text simplification tool for readers with different educational levels.

The current tool has several limitations that will be addressed in the future. The most critical limitation is that the sentence level suggestions, which focus on grammar and sentence construction, are not tuned for the topic of the text. Figure 2 shows a case where the rule example topic (teeth/dental) is fairly different than the text topic (cirrhosis). Given the wide range of topics, text-specific examples are unlikely to always be found, but we hope to present examples more closely related to the topic. The lack of existing large medical corpora to generate such specific examples specific to the topic of a text lies at the basis of this limitation.

## CONCLUSION

Text simplification is a difficult task that requires the combination of different skills, some of which we can algorithmically support. With our editor, we have shown that it is possible to support writers without requiring training or extensive linguistics knowledge. With increasing improvements in machine learning end-to-end models, we believe many more improvements are on the horizon, especially when human and machine input can be combined in an efficient manner and in a user-friendly presentation format.

Our future work will address limitations mentioned above which includes improvements in the interface and the addition of more features as we discover and validate them. Besides these incremental improvements, we also are adding audio generation components to prepare text for distribution using smart speakers and other audio means.

## FUNDING

This work was supported by the National Library of Medicine of the National Institutes of Health under Award Number R01LM011975.

## AUTHOR CONTRIBUTIONS

GL and DK are responsible for design, implementation, and evaluation of the software as well as the study design, execution, writeup, and analysis. DH and DS are responsible for the study design, execution, and analysis.

## SUPPLEMENTARY MATERIAL

Supplementary material is available at *JAMIA Open* online.

## Supplementary Material

ooac044_Supplementary_Data
