# Supplementary material for: Evaluation of an online text simplification editor using manual and automated metrics for perceived and actual text difficulty
Source: JAMIA Open. 2022 May 30;5(2):ooac044. doi: 10.1093/jamiaopen/ooac044 (PMC9155254; doi:10.1093/jamiaopen/ooac044)
Supplement: ooac044_Supplementary_Data [file ooac044_supplementary_data.zip › ooac044_Supplementary_Data/Appendix A.docx]

| **Existing Guidelines** | **New Guidelines** | **Comments/Comparison with other work** |
| --- | --- | --- |
| **Readability Formulas:** |  |  |
|  |  | No correlation found with actual comprehension/difficulty; only a correlation with perceived difficulty. |
| Word length | Term Frequency (E/S) | A stand-in for term difficulty, for English the Google Web Corpus [[1](#_ENREF_1)] works well [[2-4](#_ENREF_2)], for Spanish LexEsp [[5](#_ENREF_5)] was helpful [[6](#_ENREF_6)]. |
| Sentence length | Short sentences (E/S) | Good connectors between sentences are important for flow [[7](#_ENREF_7)]. |
|  | Grammar Frequency (E/S) | A stand-in for grammar difficulty of a sentence [[8](#_ENREF_8)], but specific guidance is needed on how to simplify [[9](#_ENREF_9)]. Spanish uses more varied structures [[10](#_ENREF_10)]. |
|  | Grammar Rules | Grammar rules were extracted using a parallel corpus with expert simplifications [[11](#_ENREF_11)]. |
| **Plain language:** |  | Difficult to judge what is plain. Plain is not necessarily simple. |
| Simple words | Term Frequency (E/S) | See above. |
|  | Use more verbs and function words (E/S) | Simple text contains more verbs and function words and fewer nouns (for other parts-of-speech, see papers) [[10](#_ENREF_10)] |
|  | Definition Creation (E/S) | Based on word morphology, new definitions are suggested in English and Spanish [[12](#_ENREF_12)]. |
|  | Definition Insertion | The use of parenthesis (do you put the difficult term or the easy explanation between parentheses) is not straightforward [[13](#_ENREF_13)]. |
|  | Term specificity and ambiguity | Highly technical terms are more difficult [[14](#_ENREF_14)]. This is not implemented as a separate feature in our tool: based on sampling, frequency (a later discovered feature) take care of this problem. |
| Short sentences | Sentence length cutoff point | Based on Wikipedia comparisons, most simplified text used shorter sentences [[15](#_ENREF_15)]. |
| **Other Advice:** |  |  |
| Short noun phrases | Only 4 or more nouns should be split up | Phrasing may be more important than length. Splitting noun phrases was only effective for very long phrases + when certain functions words were used + if it sounded more natural to a native speaker [[16](#_ENREF_16)]. |
| No double negative | Simplified negation | Three types of negation are identified by our parser and online editor [[17](#_ENREF_17)]. |
| Logical content. | Limit mixing of different topics | Topics are represented by lexical chains. Several features based on lexical chains matter; however, crossing lexical chains is the most important [[18](#_ENREF_18)]. |
|  | Missing grammatical elements | Make connections in text explicit (E/S) (audio) (paper ready for submission). |
| **Tools:** |  |  |
| Training required.  No concrete suggestions | Online editor | Our tool combines our feature and identification and translation algorithms [[19](#_ENREF_19), [20](#_ENREF_20)]. |
| Suggestions of next words | Automation – Sentence Completion | Algorithms using deep learning to suggestion simple words during the writing process (ready for submission) [[21](#_ENREF_21)] |

1. Brants, T. and A. Franz. *The google web 1T 5-gram corpus version 1.1*. Accessed 2012; Available from: <http://www.ldc.upenn.edu/Catalog/docs/LDC2006T13/readme.txt>.

2. Leroy, G. and J.E. Endicott. *Term Familiarity to Indicate Perceived and Actual Difficulty of Text in Medical Digital Libraries*. in *International Conference on Asia-Pacific Digital Libraries (ICADL 2011) - Digital Libraries -- for Culture Heritage, Knowledge Dissemination, and Future Creation*. 2011. Beijing, China.

3. Leroy, G., et al., *User Evaluation of the Effects of a Text Simplification Algorithm using Term Familiarity on Perception, Understanding, Learning and Information Retention.* Journal of Medical Internet Research (JMIR), 2013. **15**(7): p. e144 (doi:10.2196/jmir.2569).

4. Leroy, G. and D. Kauchak, *The Effect of Word Familiarity on Actual and Perceived Text Difficulty.* Journal of the American Medical Informatics Association, 2014.

5. Nurian Sebastián Gallés (Universitat de Barcelona), *LexEsp: Léxico informatizado del español*, 2000: <http://www.psico.uniovi.es/Dpto_Psicologia/metodos/soft/corpus/>.

6. Leroy, G., et al., *Spanish Text Simplification Using Term Familiarity: Applying Principles from English Text Simplification*, in *AMIA Fall Symposium*2017: Washington DC.

7. Kauchak, D., et al., *Predicting Transition Words between Sentences for English and Spanish Medical Text*, in *AMIA Fall Symposium*, AMIA, Editor 2019: Washington DC.

8. Kauchak, D., G. Leroy, and A. Hogue, *Measuring Text Difficulty Using Parse-Tree Frequency.* Journal of American Society of Information Science and Technology (JASIST), 2017. **68**(9): p. 2088-2100.

9. Kauchak, D., G. Leroy, and M. Just, *Grammar Frequency and Simplification: When Intuition Fails*, in *AMIA Fall Symposium*2016: Chicago.

10. Mukherjee, P., et al., *The Role of Surface, Semantic and Grammatical Features on Simplification of Spanish Medical Texts: A User Study*, in *AMIA Fall Symposium*, AMIA, Editor 2017: Washington DC.

11. Szep, A., et al., *Algorithmic Generation of Grammar Simplification Rules Using Large Corpora*, in *AMIA Summit*, AMIA, Editor 2019: San Francisco.

12. Kloehn, N., et al., *SubSimplify - Automatically Generating Term Explanations in English and Spanish when Expert and Big Data Dictionaries are Insufficient.* Journal of Medical Internet Research (JMIR), 2018. . **20**(8).

13. Gu, Y., G. Leroy, and D. Kauchak, *When synonyms are not enough: Optimal parenthetical insertion for text simplification*, in *AMIA Fall Symposium*2017: Washington DC.

14. Kauchak, D., et al., *Text Simplification Tools: Using Machine Learning to Discover Features that Identify Difficult Text*, in *47th Hawaii International Conference on System Sciences (HICSS)*2014: Waikaloa, Big Island,Hawaii. p. 2616-2625.

15. Pei, M., et al., *Splitting Sentences for Text Simplification: A Machine Learning Approach*, in *AMIA Summit*, AMIA, Editor 2019: San Francisco.

16. Leroy, G., D. Kauchak, and A. Hogue, *Effects of Text Simplification: Evalution of Splitting up Noun Phrases.* Journal of Health Communication: International Perspectives, 2016. **21**(S1): p. 18-26.

17. Mukherjee, P., et al., *NegAIT: A New Parser for Medical Text Simplification Using Morphological, Sentential and Double Negation.* Journal of Biomedical Informatics, 2017. **69**: p. 55–62.

18. Mukherjee, P., G. Leroy, and D. Kauchak, *Using Lexical Chains to Identify Text Difficulty: A Corpus Statistics and Classification Study.* IEEE Journal of Biomedical and Health Informatics, 2018. **23**(5): p. 2164 - 2173 (PMID: 30530380).

19. Kauchak, D. and G. Leroy, *A Web-Based Medical Text Simplification Tool*, in *Hawaii International Conference on System Sciences (HICSS)*2020: Maui, Hawaii.

20. Kauchak, D., G. Leroy, and M. Grueter, *Demo: An Online Evidence-based Text Simplification Editor for Medical Text*, in *Workshop on Information Technology and Systems (WITS)*2018: Santa Clara. p. [Demo].

21. Hung, H.N., D. Kauchak, and G. Leroy, *AutoMeTS: The Autocomplete for Medical Text Simplification*, in *28th International Conference on Computational Linguistics, COLING*Under Review: Online.
