# Supplementary material for: Evaluation of an online text simplification editor using manual and automated metrics for perceived and actual text difficulty
Source: JAMIA Open. 2022 May 30;5(2):ooac044. doi: 10.1093/jamiaopen/ooac044 (PMC9155254; doi:10.1093/jamiaopen/ooac044)
Supplement: ooac044_Supplementary_Data [file ooac044_supplementary_data.zip › ooac044_Supplementary_Data/Appendix B - Text and Questions.docx]

**APPENDIX B: TEXT AND QUESTIONS**

**TEXT: ASTHMA**

**True/False Questions before Reading the Text**

- The severity of asthma is determined by multiple tests. [[true]]
- Taking asthma medication for prolonged periods can make asthma worse. [[false]]
- Having hay fever is a main cause of asthma in many people. [[false]]
- Research has shown that asthma is linked to obesity. [[true]]
- Corticosteroids are the only effective inhaled medication for asthma. [[false]]

**Original Text**

Asthma (from the Greek άσθμα, ásthma, "panting") is the common chronic inflammatory disease of the airways characterized by variable and recurring symptoms, reversible airflow obstruction, and bronchospasm. Symptoms include wheezing, coughing, chest tightness, and shortness of breath. Asthma is clinically classified according to the frequency of symptoms, forced expiratory volume in 1 second (FEV1), and peak expiratory flow rate. Asthma may also be classified as atopic (extrinsic) or non-atopic (intrinsic).

It is thought to be caused by a combination of genetic and environmental factors. Treatment of acute symptoms is usually with an inhaled short-acting beta-2 agonist (such as salbutamol). Symptoms can be prevented by avoiding triggers, such as allergens and irritants, and by inhaling corticosteroids. Leukotriene antagonists are less effective than corticosteroids and thus less preferred.

Its diagnosis is usually made based on the pattern of symptoms and/or response to therapy over time. The prevalence of asthma has increased significantly since the 1970s. As of 2010, 300 million people were affected worldwide. In 2009 asthma caused 250,000 deaths globally. Despite this, with proper control of asthma with step down therapy, prognosis is generally good.

Causes

Asthma is caused by environmental and genetic factors. These factors influence how severe asthma is and how well it responds to medication. The interaction is complex and not fully understood.

Studying the prevalence of asthma and related diseases such as eczema and hay fever have yielded important clues about some key risk factors. The strongest risk factor for developing asthma is a history of atopic disease; this increases one's risk of hay fever by up to 5× and the risk of asthma by 3–4×. In children between the ages of 3–14, a positive skin test for allergies and an increase in immunoglobulin E increases the chance of having asthma. In adults, the more allergens one reacts positively to in a skin test, the higher the odds of having asthma.

Because much allergic asthma is associated with sensitivity to indoor allergens and because Western styles of housing favor greater exposure to indoor allergens, much attention has focused on increased exposure to these allergens in infancy and early childhood as a primary cause of the rise in asthma. Primary prevention studies aimed at the aggressive reduction of airborne allergens in a home with infants have shown mixed findings. Strict reduction of dust mite allergens, for example, reduces the risk of allergic sensitization to dust mites, and modestly reduces the risk of developing asthma up until the age of 8 years old. However, studies also showed that the effects of exposure to cat and dog allergens worked in the converse fashion; exposure during the first year of life was found to reduce the risk of allergic sensitization and of developing asthma later in life.

The inconsistency of this data has inspired research into other facets of Western society and their impact upon the prevalence of asthma. One subject that appears to show a strong correlation is the development of asthma and obesity. In the United Kingdom and United States, the rise in asthma prevalence has echoed an almost epidemic rise in the prevalence of obesity. In Taiwan, symptoms of allergies and airway hyper-reactivity increased in correlation with each 20% increase in body-mass index. Several factors associated with obesity may play a role in the pathogenesis of asthma, including decreased respiratory function due to a buildup of adipose tissue (fat) and the fact that adipose tissue leads to a pro-inflammatory state, which has been associated with non-eosinophilic asthma.

Asthma has been associated with Churg–Strauss syndrome, and individuals with immunologically mediated urticaria may also experience systemic symptoms with generalized urticaria, rhino-conjunctivitis, orolaryngeal and gastrointestinal symptoms, asthma, and, at worst, anaphylaxis. Additionally, adult-onset asthma has been associated with periocular xanthogranulomas.

**Simplified Text**

Asthma (from the Greek word for "panting," which means rapid shallow breathing) is the common chronic inflammatory condition of the airways characterized by recurring symptoms, blocking of the air flow, and spasming of the airways (bronchospasms). Symptoms include wheezing, coughing, tightening of the chest, and shortness of breath. The severity of asthma is determined based on how often symptoms happen and the results of two tests: 1) the amount of air blown in 1 second (FEV1), and the strongest amount of air that can be blown. Asthma may also be classified as atopic (outside) or non-atopic (inside).

Asthma is thought to be caused by a combination of genetic and environmental factors. Treatment of is usually an inhaled medicine (such as salbutamol). Symptoms can be prevented by avoiding triggers, such as allergens and irritants, and by inhaling medicine (for example, corticosteroids). For medicine, corticosteroids are more effective than leukotriene antagonists.

Diagnosis of asthma is usually made based on the pattern of symptoms and/or response to therapy over time. The number of people with asthma has increased significantly since the 1970s. As of 2010, 300 million people had it worldwide. In 2009, 250,000 worldwide died of asthma. However, with proper treatment, outcomes are generally good.

Causes

Asthma is caused by both the environment and genes. The cause determines how serious asthma is and how well it responds to medicine. How the genes and environment interact is complex and not fully understood.

Studying the prevalence of asthma and related conditions such as eczema and hay fever have given important clues about some key risk factors. The strongest risk factor for developing asthma is a history of reacting to things in the environment. The risk of having hay fever increases by up to 5 times and the risk of asthma by 3–4 times. In children between the ages of 3 to 14, a positive skin test for allergies and an increase in immunoglobulin E increases the chance of getting asthma. In adults, the more allergens one reacts to in a skin test, the higher the odds of having asthma.

Allergic asthma is linked with sensitivity to indoor allergens, and Western styles of housing have more indoor allergens. Research has focused on increased exposure to these allergens when people are young as a main cause of the increases in asthma. Studies aimed at preventing asthma looked at whether greatly reducing airborne allergens in a home with babies have shown mixed findings. Reducing dust mite allergens, for example, cut down the risk of allergy to dust mites, and modestly cut the risk of developing asthma up until the age of 8 years old. However, studies also showed that exposure to cats and dogs was beneficial. Exposure during the first year of life to cats and dogs was found to cut down the risk of allergic sensitization and of developing asthma later in life.

The inconsistency of this data has resulted in research into other parts of Western society and their impact upon the prevalence of asthma. One subject that seems to correlate strongly is the development of asthma and obesity. In the United Kingdom and United States, the increase in asthma has mirrored the increase in obesity. In Taiwan, symptoms of allergies and asthma increased with each 20% increase in body-mass index. Several factors linked with obesity may play a part in the beginning of asthma, including reduced respiratory function due to a buildup of fat and the fact that fatty tissue results in a pro-inflammatory state, which has been linked with asthma.

Asthma has been linked with Churg–Strauss syndrome, and people who get hives may also experience other symptoms: rhino-conjunctivitis, orolaryngeal and gastrointestinal symptoms, asthma, and, at worst, allergic shock. Adult asthma has been linked with periocular xanthogranulomas.

**Multiple-choice Questions Presented with the Text**

(perceived difficulty) After reading this text, I consider this information:

- Very difficult to understand
- Difficult to understand
- Easy to understand
- Very easy to understand

(Overview question) This text provides information on risk factors for asthma:

- No, it mainly discusses the different causes of asthma.
- No, it mainly discusses different types of asthma
- Yes, it discusses allergens as risk factors. [[answer]]
- Yes, it discusses childhood presence of pets as risk factors.

(General question) Asthma is caused

- by environmental and genetic factors. [[answer]]
- mostly by environmental factors.
- mostly by genetic factors.
- by triggers in the environment.

**Multiple-choice Questions Presented after Reading the Text**

Asthma symptoms include all of the following *except*:

1. coughing.
2. slow breathing. [[answer]]
3. wheezing.
4. tightening in the chest.

How are asthma symptoms most effectively avoided?

1. by avoiding triggers and using inhaled medication. [[answer]]
2. by using inhaled medication.
3. by using inhaled medication once symptoms occur.
4. by avoiding triggers.

Which of the following is a common measure for diagnosing asthma?

1. the amount of air you can breathe in in one second.
2. the amount of air you can breathe out in one second. [[answer]]
3. the amount of air you can breathe in in one breath.
4. the amount of air you can breathe out in one breath.

Which of the following is NOT related to developing asthma?

1. obesity.
2. hay fever. [[answer]]
3. exposure to dogs and cats at a young age.
4. dust mite allergens at a young age.

**Repeat True/False Questions Presented after Reading the Text**

- The severity of asthma is determined by multiple tests. [[true]]
- Taking asthma medication for prolonged periods can make asthma worse. [[false]]
- Having hay fever is a main cause of asthma in many people. [[false]]
- Research has shown that asthma is linked to obesity. [[true]]
- Corticosteroids are the only effective inhaled medication for asthma. [[false]]

**Text: Liver cirrhosis**

**True/False Questions before Reading the Text**

- Liver cirrhosis can be cured if caught very early on. [[false]]
- Liver cirrhosis can be caused by alcoholism or hepatitis. [[true]]
- The liver is an essential organ because it generates red blood cells. [[false]]
- People with liver cirrhosis can have both scars and fibrous tissue in the liver. [true]
- A visual inspection of the liver using laparoscopy is commonly used for diagnosis. [[false]]

**Original Text**

Cirrhosis is a consequence of chronic liver disease characterized by replacement of liver tissue by fibrosis, scar tissue and regenerative nodules (lumps that occur as a result of a process in which damaged tissue is regenerated), leading to loss of liver function. Cirrhosis is most commonly caused by alcoholism, hepatitis B and C, and fatty liver disease, but has many other possible causes. Some cases are idiopathic (i.e., of unknown cause).

Ascites (fluid retention in the abdominal cavity) is the most common complication of cirrhosis, and is associated with a poor quality of life, increased risk of infection, and a poor long-term outcome. Other potentially life-threatening complications are hepatic encephalopathy (confusion and coma) and bleeding from esophageal varices. Cirrhosis is generally irreversible, and treatment usually focuses on preventing progression and complications. In advanced stages of cirrhosis, the only option is a liver transplant.

The word "cirrhosis" derives from Greek κιρρός [kirrhós] meaning yellowish, tawny (the orange-yellow colour of the diseased liver) + Eng. med. suff. -osis. While the clinical entity was known before, it was René Laennec who gave it the name "cirrhosis" in his 1819 work in which he also describes the stethoscope.

Pathophysiology

The liver plays a vital role in synthesis of proteins (e.g., albumin, clotting factors and complement), detoxification and storage (e.g., vitamin A). In addition, it participates in the metabolism of lipids and carbohydrates.

Cirrhosis is often preceded by hepatitis and fatty liver (steatosis), independent of the cause. If the cause is removed at this stage, the changes are still fully reversible.

The pathological hallmark of cirrhosis is the development of scar tissue that replaces normal parenchyma, blocking the portal flow of blood through the organ and disturbing normal function. Recent research shows the pivotal role of the stellate cell, a cell type that normally stores vitamin A, in the development of cirrhosis. Damage to the hepatic parenchyma leads to activation of the stellate cell, which becomes contractile (called myofibroblast) and obstructs blood flow in the circulation. In addition, it secretes TGF-β1, which leads to a fibrotic response and proliferation of connective tissue. Furthermore, it secretes TIMP 1 and 2, naturally occurring inhibitors of matrix metalloproteinases, which prevents them from breaking down fibrotic material in the extracellular matrix.

The fibrous tissue bands (septa) separate hepatocyte nodules, which eventually replace the entire liver architecture, leading to decreased blood flow throughout. The spleen becomes congested, which leads to hypersplenism and increased sequestration of platelets. Portal hypertension is responsible for most severe complications of cirrhosis.

Diagnosis

The gold standard for diagnosis of cirrhosis is a liver biopsy, through a percutaneous, transjugular, laparoscopic, or fine-needle approach. A biopsy is not necessary if the clinical, laboratory, and radiologic data suggests cirrhosis. Furthermore, there is a small but significant risk to liver biopsy, and cirrhosis itself predisposes for complications due to liver biopsy. Ascites, low platelet count, and spider nevi are useful physical findings.

**Simplified text:**

Cirrhosis is a result of chronic liver disease that leads to the liver not being able to work correctly. Cirrhosis replaces healthy liver tissue with fibrosis, scar tissue, and lumps from regrown damaged tissue (regenerative nodules). Cirrhosis is usually caused by alcohol abuse, hepatitis B and C, and fatty liver disease, but has many other causes. Some cases do not have a known cause (called, idiopathic cases).

Fluid build up in the abdomen (ascites) is the most common complication of cirrhosis, and is linked with a poor quality of life, increased risk of infection, and a poor long-term outcome. Other potentially life-threatening complications are confusion and coma (hepatic encephalopathy) and bleeding from varicose veins in the esophagus. Cirrhosis cannot be reversed, and treatment usually focuses on preventing the cirrhosis from getting worse and developing complications. In advanced stages of cirrhosis, the only choice is a liver transplant.

The word "cirrhosis" comes from the Greek word kirrhós, meaning yellowish or tawny (the orange-yellow color of the diseased liver). While the disease was known before, it was René Laennec who gave it the name "cirrhosis" in 1819 in a work where he also describes the stethoscope.

Pathophysiology

The liver is critical in making proteins (e.g., albumin, clotting factors and complement), removing toxins from the blood, and storing nutrients (e.g., vitamin A). It also takes part in the processing of fats (lipids) and sugars (carbohydrates).

No matter what the cause, hepatitis and fatty liver (steatosis) are usually present before the liver develops cirrhosis. If the cause of the hepatitis or fatty liver is removed at this stage, the changes to the liver can still be reversed.

The best indicator of cirrhosis is the development of scar tissue that replaces healthy liver tissue, blocking the flow of blood through the liver and keeping it from working normally. Recent research shows the important role of the stellate cell, a cell type that normally stores vitamin A, in the development of cirrhosis. Damage to the healthy liver tissue triggers the stellate cell, which becomes contracted (called myofibroblast) and blocks blood flow in the circulation. In addition, it secretes TGF-β1, which leads to a an over-production of connective tissue (healthy tissue is replaced by tissue that can't do the same functions). What is more, it secretes TIMP 1 and 2, naturally occurring inhibitors of matrix metalloproteinases, which prevents them from breaking down fibrotic material in the structure around the cells.

The fibrous tissue bands (septa) separate liver cell nodules, which eventually replace the entire liver structure, leading to decreased blood flow throughout. The spleen becomes congested, which leads to an enlarged spleen and increased separation of platelets. Portal hypertension (high blood pressure in the vein to the liver) is responsible for the worst complications of cirrhosis.

Diagnosis

Cirrhosis is best diagnosed with a liver biopsy. A biopsy is not necessary if the clinical, laboratory, and radiologic data indicate cirrhosis. Furthermore, there is a small but significant risk to liver biopsy, and cirrhosis itself predisposes for complications due to liver biopsy. Ascites, low platelet count, and moles with spider-like veins are other useful physical findings.

**Multiple-choice Questions Presented with the Text**

(Perceived Difficulty) After reading this text, I consider this information

- Very difficult to understand
- Difficult to understand
- Easy to understand
- Very easy to understand

(Overview Question) This text explains

- Different treatment options for liver cirrhosis versus hepatitis.
- Different treatment options developed for liver cirrhosis over the last century.
- Effects of liver cirrhosis on the liver [[answer]]
- Effects of liver cirrhosis on blood cell counts.

(General question) Which of the following is a complication of liver cirrhosis?

- Retaining too many fluids in the body, which increases the chance of infections [[answer]]
- Retaining too much fat, resulting in fatty liver disease
- Not being able to retain any or enough Vitamin A
- Weakness of the esophageal veins leading to a contamination of the blood

**Multiple-choice Questions Presented after Reading the Text**

How are liver cirrhosis and hepatitis related?

- Hepatitis is a disease that occurs often before cirrhosis and directly causes it. [[answer]]
- Hepatitis is a disease that occurs often as a result of cirrhosis and is caused by it.
- Hepatitis is a blood disease that indirectly causes cirrhosis by preventing the absorption of vitamin A.
- Hepatitis is a cell disease that indirectly causes cirrhosis by changing the shape of stellate cells.

Why is blood flow in the liver an important problem with liver cirrhosis?

- Scar tissue develops, which requires new veins to be formed.
- There is a multiplication of connective veins which requires more blood flow.
- Both scar formation and changes in liver cells lead to reduced blood flow. [[answer]]
- Scar tissue develops which reduces the number of working veins and leads to reduced blood flow.

What is a complication of liver cirrhosis?

- increased blood pressure in the veins leading to the liver [[answer]]
- toxic waste in the blood
- reduced vision and yellowing of the eyes
- a contraction of the veins leading from the liver

Why is the formation of scar tissue an important complication of liver cirrhosis?

- Scar tissue replaces parts of the liver and activates stellate cells both of which lead to the blocking of blood flow through the liver [[answer]]
- Scar tissue leads to obstructions in veins which leads to blocked blood flow
- Fibrous veins are replaced by scar tissue which leads to blocked blood flow
- Scar tissue hinders TIMP1 and 2 in breaking down fibrotic cells leading to reduced blood flow

**Repeat True/False Questions Presented after Reading the Text**

- Liver cirrhosis can be cured if caught very early on. [[false]]
- Liver cirrhosis can be caused by alcoholism or hepatitis. [[true]]
- The liver is an essential organ because it generates red blood cells. [[false]]
- People with liver cirrhosis can have both scars and fibrous tissue in the liver. [true]
- A visual inspection of the liver using laparoscopy is commonly used for diagnosis. [[false]]

**Pemphigus**

**True/False Questions before Reading the Text**

- An auto-immune disorder is discovered by blisters on the skin. [[false]]
- In pemphigus vulgaris, the sores often develop on the inside of the mouth. [[true]]
- Pemphigus is triggered by repeated over-exposure to the sun. [[false]]
- Pemphigus can be treated. [[true]]
- Pemphigus vulgaris is a general term for a group of diseases such as pemphigoid, pemphigitis, etc. [[false]]

**Original Text**

Pemphigus is a general term for a group of rare autoimmune blistering skin disorders. Autoimmune disorders occur when the body's own immune system mistakenly attacks healthy tissue. The symptoms and severity associated with the various forms of pemphigus vary. All forms of pemphigus are characterized by the development of blistering eruptions on the outer layer of the skin (epidermis). In pemphigus vulgaris, lesions also develop on the mucous membranes such as those lining the inside the mouth. Mucous membranes are the thin, moist coverings of many of the body's internal surfaces. If left untreated, pemphigus will usually be fatal. The exact cause of pemphigus is unknown.

The term pemphigus is a general term for a group of related autoimmune blistering skin diseases. The two main types of pemphigus are pemphigus vulgaris and pemphigus foliaceus. Each type has subtypes. Additional disorders are sometimes classified as pemphigus including paraneoplastic pemphigus and pemphigus IgA. Some physicians consider these disorders similar, yet distinct, autoimmune blistering disorders with different causes and clinical, immunological and microscopic tissue (histological) features. Pemphigoid is a general term for a different group of skin disorders. These other disorders are discussed in the related disorders section of this report.

**Simplified Text**

Pemphigus is a group of rare autoimmune blistering skin conditions. Autoimmune conditions come about when the body's own immune system mistakenly attacks healthy tissue. The many kinds of pemphigus have different symptoms and intensity. All kinds of pemphigus get blisters on the top layer of the skin (epidermis). In pemphigus vulgaris, blisters also develop on the mucus tissue layer like those lining the inside the mouth. Mucus tissue layers are the thin, wet natural coverings of many of the body's inside surfaces. If not treated, patients with pemphigus will usually die. The exact cause of pemphigus is not known.

The two main types of pemphigus are pemphigus vulgaris and pemphigus foliaceus. Each type has subtypes. Additional conditions that are included by some doctors as pemphigus include paraneoplastic pemphigus and pemphigus IgA. These conditions also are autoimmune blistering conditions, though they have different causes and clinical, immunological, and microscopic tissue (histological) features. Pemphigoid is a general term for a different group of skin conditions. These other conditions are talked about in the related conditions section of this report.

**Multiple-choice Questions Presented with the Text**

(Perceived Difficulty) After reading this text, I consider this information

- Very difficult to understand
- Difficult to understand
- Easy to understand
- Very easy to understand

(Overview Question) This text is about autoimmune disorders:

- No, it is about blisters.
- No, it is about membranes
- Yes, it is about autoimmune disorders resulting in blisters. [[correct]]
- Yes, it is about autoimmune disorders resulting in membrane mucus.

(General Question) There are two main types of pemphigus

- Yes, pemphigus vulgaris and pemphigus foliaceus [[correct]]
- Yes, pemphigus vulgaris and pemphigoid
- Yes, pemphigus foliaceus and pemphigoid
- No, there are three main types: pemphigus vulgaris, pemphigus foliaceus and pemphigoid

**Multiple-choice Questions Presented after Reading the Text**

With pemphigus vulgaris, blisters will develop:

- On the outside of the body
- On the inside of the body
- Both outside and inside of the body. [Answer]
- The text does not contain enough information to answer the question.

Pemphigus is

- a reaction to lesions in the skin
- a condition where we cannot define the cause [Answer]
- a reaction to microscopic tissue features
- has a consistent set of symptoms

Pemphigus is a condition

- where the body tries to damage healthy tissue because it thinks something is wrong with it. [answer]
- where the body tries to replace healthy tissue because it thinks something is wrong with it.
- where the body tries to protect itself against tissue not from the person’s own body
- where the body tries to protect itself against tissue that has blisters

The different types of pemphigus are well established.

- Yes, the different types of blistering allows for differentiation
- Yes, the causes are clear and allow for differentiation
- No, some related disorders are sometimes classified as pemphigus [answer]
- No, some unknown blistering conditions are sometimes classified as pemphigus

**Repeat True/False Questions Presented after Reading the Text**

- An auto-immune disorder is discovered by blisters on the skin. [[false]]
- In pemphigus vulgaris the sores often develop on the inside of the mouth. [[ true]]
- Pemphigus is usually caused by repeated over-exposure to the sun. [[false]]
- Pemphigus can be treated. [[true]]
- Pemphigus vulgaris is a general term for a group of diseases such as pemphigoid, pemphigitis, etc. [[false]]

**TEXT: POLYCYTHEMIA VERA**

**True/False Questions before Reading the Text**

- Polycythemia vera does not result in an increase in white blood cells. [[false]]
- Most of the people affected by polycythemia have a genetic mutation.[[true]]
- Hyperviscosity is a condition where a person's blood becomes too thin. [[false]]
- Polycythemia causes a person to produce too many red blood cells. [[true]]
- Polycythemia has only a single symptom. [[false]]

**Original Text**

Polycythemia vera is a rare, chronic disorder involving the overproduction of blood cells in the bone marrow (myeloproliferation). The overproduction of red blood cells is most dramatic. But the production of white blood cells and platelets are also elevated in most cases. Since red blood cells are overproduced in the marrow, this leads to abnormally high numbers of circulating red blood cells (red blood mass) within the blood. Consequently, the blood thickens and increases in volume, a condition called hyperviscosity. Thickened blood may not flow through smaller blood vessels properly. A variety of symptoms can occur in individuals with polycythemia vera including nonspecific symptoms such as headaches, fatigue, weakness, dizziness or itchy skin; an enlarged spleen (splenomegaly); a variety of gastrointestinal issues; and the risk of blood clot formation, which may prevent blood flow to vital organs. More than 90 percent of individuals with polycythemia vera have a mutation of the JAK2 gene. The exact role of this mutation in the development of polycythemia vera is not yet known.

Polycythemia vera belongs to a group of diseases known as the myeloproliferative disorders (MPDs). Three other disorders are commonly classified as MPDs: chronic myeloid leukemia, essential thrombocythemia and idiopathic myelofibrosis.

**Simplified Text**

Polycythemia vera is a rare, chronic condition where the bone marrow makes too many blood cells (myeloproliferation). Red blood cells increase the most, but white blood cells and platelets are also high in most cases. The increase in red blood cells in the bone marrow results in too many red blood cells (red blood mass) in the blood. This causes the blood to thicken and increase in volume causing the blood to be too thick to flow through smaller blood vessels, a condition called hyperviscosity. Someone with polycythemia vera may have many different symptoms, including symptoms that are not specific such as headaches, lack of energy, loss of strength, spinning sensation, or itchy skin; an enlarged spleen (splenomegaly); stomach and intestinal issues; and the risk of forming blood clots, which may prevent blood flow to critical organs. More than 90 percent of people with polycythemia vera have a mutation of the JAK2 gene. The exact function of this mutation in the development of polycythemia vera is not yet known.

Polycythemia vera belongs to a group of conditions known as the myeloproliferative disorders (MPDs). Three other disorders are commonly classified as MPDs: chronic myeloid leukemia, essential thrombocythemia and idiopathic myelofibrosis.

**Multiple-choice Questions Presented with the Text**

(Perceived Difficulty) After reading this text, I consider this information

- Very difficult to understand
- Difficult to understand
- Easy to understand
- Very easy to understand

(Overview Question) This text is about a disorder affecting bone marrow

- No, it is about a blood disorder.
- No, it is about rare cancer
- Yes, it is about disorders affecting blood cells in bone marrow. [answer]
- Yes, it is about disorders affecting nerve tissue in bone marrow.

(General Question) Polycythemia vera is

- One of four disorders in the group of myeloproliferative disorders [answer]
- The main disorder with 3 subcategories in the group of myeloproliferative disorders
- A disorder often misclassified in the group of myeloproliferative disorders
- A disorder that is a subcategory of idiopathic myelofibrosis

**Multiple-choice Questions Presented after Reading the Text**

Polycythemia vera is a blood disease where

- too many white blood cells are around which makes the bone marrow produce too many red blood cells in response.
- too many red blood cells are around leaving not enough room for white blood cells and platelets.
- too many red blood cells are around and this makes the blood too thick [answer]
- too many red and white blood cells and platelets are around and they start working against each other.

People with polycythemia vera make

- more red and white blood cells equally
- more red and white blood cells with even more red blood cells [answer]
- less red and white blood cells equally
- more bone marrow

A known symptom of the disorder is that

- the blood may form clots that can stop it from reaching essential organs [answer]
- the arteries start narrowing so that the blood cannot get to the heart
- the JAK2 gene malfunctions
- too many red blood cells start canceling the effect of white blood cells

A person with polycythemia vera can suffer from many different symptoms

- that are all fairly specific to the disease, such as blood clots
- that are frequently found with other diseases, such as headaches
- that are a mix of those easily found with other diseases, such as stomach pains and tiredness [answer]
- that can be easily recognized by checking the narrowing of the veins

**Repeat True/False Questions Presented after Reading the Text**

- Polycythemia vera does not result in an increase in white blood cells. [[false]]
- Most of the people affected by polycythemia have a genetic mutation. [[true]]
- Hyperviscosity is a condition where a person's blood becomes too thin. [[false]]
- Polycythemia causes a person to produce too many red blood cells. [[true]]
- Polycythemia has only a single symptom. [[false]]
